# Effects of concentration of amyloid β (Aβ) on viability of cultured retinal pigment epithelial cells

**DOI:** 10.1186/s12886-019-1076-3

**Published:** 2019-03-08

**Authors:** Naonori Masuda, Hiroki Tsujinaka, Hiromasa Hirai, Mariko Yamashita, Tetsuo Ueda, Nahoko Ogata

**Affiliations:** Department of Ophthalmology, Nara Medical University, 840 Shijo-cho, Kashihara, 634-8522 Japan

**Keywords:** Age-related macular degeneration, Amyloid beta, Retinal pigment epithelial cells, Vascular endothelial growth factor, Pigment epithelium-derived factor, Receptor for advanced glycation end products

## Abstract

**Background:**

Amyloid beta (Aβ) is a constituent of drusen that is a common sign of age-related macular degeneration (AMD). The purpose of this study was to investigate the effect of Aβ on human retinal pigment epithelial (RPE) cells in culture.

**Methods:**

Cells from a human RPE cell line (ARPE-19) were exposed to 0 to 25 μM of Aβ 1–40 for 48 h, and the number of living cells was determined by WST-8 cleavage. Replicative DNA synthesis was measured by the incorporation of 5′-bromo-2′-deoxyuridine. The cell death pathway was investigated by the WST-8 cleavage assay after the addition of caspase-9 inhibitor, an anti-apoptotic factor. Real-time qRT-PCR was performed using Aβ-exposed cellular RNA to determine the level of vascular endothelial growth factor (VEGF)-A and pigment epithelium derived factor (PEDF). To determine the effect of receptor-for-advanced glycation end products (RAGE), the siRNA for RAGE was inserted into ARPE-19 treated with Aβ, and the levels of expression of *VEGF-A* and *PEDF* were determined.

**Results:**

The number of living ARPE-19 cells was increased by exposure to 5 μM Aβ but was decreased by exposure to 25 μM of Aβ. Replicative DNA synthesis by ARPE-19 cells exposed to 25 μM of Aβ was significantly decreased indicating that 25 μM of Aβ inhibited cell proliferation. Real-time RT-PCR showed that the level of the mRNA of *PEDF* was increased by exposure to 5 μM Aβ, and the levels of the mRNAs of *PEDF* and *VEGF-A* were also increased by exposure to 25 μM Aβ. The addition of an inhibitor of caspase-9 blocked the decrease the number of ARPE-19 cells exposed to 25 μM Aβ. Exposure to si-RAGE attenuated the increase of *VEGF-A* and *PEDF* mRNA expression in ARPE-19 exposed to Aβ.

**Conclusions:**

Exposure of ARPE-19 cells to low concentrations of Aβ increases the level of PEDF which then inhibits the apoptosis of ARPE-19 cells leading to RPE cell proliferation. Exposure to high concentrations of Aβ induces RPE cell death and enhances the expression of the mRNA of VEGF-A in RPE cells. The Aβ-RAGE pathway may lead to the expression *VEGF-A* and *PEDF* in RPE cells. These results suggest that Aβ is strongly related to the pathogenesis of choroidal neovascularization.

## Background

Age-related macular degeneration (AMD) is a progressive eye disorder that can proceed to irreversible blindness [1]. Genetic and environmental factors are known to modify the visual reduction although the relative importance of each of the factors has not been definitively determined. It has been established that one of the strongest predictors for the development of AMD is the number and size of drusen [2]. Drusen are small, yellowish-white protuberant lesions seen in the fundus ophthalmoscopically and are considered to be the initial change in eyes with AMD.

In spite of the well-known relationship between drusen and AMD, there are still some unanswered questions, e.g., how drusen form and how drusen interact with the RPE and photoreceptor cells. It was recently reported that amyloid beta (Aβ), a peptide associated with the neurodegenerative events in Alzheimer’s disease, is an important constituent of drusen [3]. Aβ is a toxic protein that affects nerve cells and is associated with many neurodegenerative disorders [4].

In the human eye, several Aβ reservoirs have been found in the retina. Elevated Aβ levels have been found in aged retinas, and Aβ has been shown to be associated with the progression of AMD [5]. Aβ 1–40 is the one of the most common and relatively less toxic form of Aβ [6]. Isas et al. reported that the concentration of Aβ1–40 is the highest among the different forms of Aβ in drusen deposits, [7] and some studies have shown how Aβ 1–40 interacts with RPE cells. For example, Liu et al. demonstrated that chronic inflammation that is an important pathogenic process in AMD is caused by the stimulation of Aβ1–40 via NF-κB activation [8], and they also demonstrated that the inflammasomes that are activated by Aβ1–40 are responsible for the upregulation of the IL-6, TNF-α, IL-1β, IL-18, caspase-1, NLRP3, and XAF1 genes in the RPE, choroid, and the neuroretina [9]. Although such findings imply that Aβ 1–40 is a potential triggering peptide for AMD, the effect of Aβ 1–40 on RPE cells remains undetermined especially on the relationship between the concentration of Aβ 1–40 and toxicity. Its effects on RPE cells need to be understood to determine its role in the development of AMD.

Thus, the purpose of this study was to determine the effects of Aβ 1–40 on human adult RPE (ARPE) cells in culture. To accomplish this, we exposed cells from an ARPE cell line to different concentrations of Aβ 1–40 and determined the number of living ARPE cells. We also examined the pathways that might be involved in the Aβ 1–40 effects on the ARPE cells.

## Methods

### Cell cultures

ARPE-19 cells (ATCC® CRL-2302™), a human RPE cell line [10], were obtained from American Type Culture Collection (Manassas, VA, USA). The cells were maintained in Dulbecco’s modified Eagles medium (Gibco®, Life Technologies, Carlsbad, CA) and F12 medium (Gibco®) 1:1 supplemented with 10% (*v*/v) fetal bovine serum (FBS), 100 units/ml penicillin G, and 100 μg/mL streptomycin (Wako Pure Chemical Industries, Ltd., Osaka, Japan). To examine the effects of Aβ on the ARPE-19 cells, several concentrations of Aβ 1–40 (Peptide Institute, Inc., Osaka, Japan) were added to the culture medium. Equivalent amount of DMSO (Wako Pure Chemical Industries), a solvent for Aβ1–40, was added to the culture medium in the control group.

### Measurements of surviving cells by water-soluble tetrazolium salt WST-8

ARPE-19 cells were seeded in 96 well plates at 1.0 × 10^4^ cells/100 μL per well. The cells were cultured with 0.5 to 25 μM of Aβ 1–40 for 48 h. After the exposure, the number of living cells was determined with the Cell Counting kit-8 (Dojindo Laboratories, Mashikimachi, Japan) according to the manufacturer’s protocol [11–13]. In brief, ARPE-19 cells were incubated with WST-8 (2-(2-methoxy-4-nitrophenyl)-3-(4-nitrophenyl)-5-(2,4-disulfophenyl)- 2H-tetrazoliummonosodium salt) solution at 37 °C for 1 to 2 h. After the exposure, the optical density at 450 nm was measured with the i-Mark™ microplate reader (Bio-Rad Laboratories, Inc., California, USA).

### Investigation of cell death pathway

To investigate the cell death pathway, ARPE-19 cells (1.0 × 10^4^ cells/100 μL/well) exposed to 5 to 25 μM of Aβ- were cultured with an anti-necroptotic factor, e.g., necrostatin-1 (50 mM) or caspase-8 (1 mM) or caspase-9 inhibitors (1 mM), for 48 h [14, 15]. After that, the number of living cells was determined by WST-8 cleavage.

### Measurement of replicative DNA synthesis

To determine the extent of cell proliferation, we assayed the degree of incorporation of thymidine analog BrdU (5′-bromo-2′-deoxyuridine) into newly synthesized DNA by using the DNA-BrdU Labeling and Detection kit (Roche Diagnostics, Mannheim, Germany) according to the manufacturer’s protocol [16, 17]. Briefly, ARPE-19 cells were cultured in 96-well plates at 1.0 × 10^4^ cells/100 μL with different concentrations of Aβ 1–40. The cells were exposed to the BrdU solution Aβ 1–40 for 48 h. After the exposure, the optical density at 450 nm was measured with the i-MarkTM microplate reader (Bio-Rad Laboratories).

### VEGF-A and PEDF messenger RNA induction

After 48 h of incubation with Aβ 1–40, the NucleoSpin RNA kit (Takara Bio, Inc., Kusatsu, Japan) was used to extract the total RNA from the ARPE-19 cells (1.0 × 10^4^ cells/100 μL per well) according to the manufacturer’s protocol. cDNAs were synthesized from the total RNA with the Exscript RT Reagent kit (Takara Bio, Inc). The PCR primers corresponding to nucleotides 2465–2536 of the mRNA of human *VEGF-A* (NM_001025366) and 489–630 for *PEDF* mRNA (NM_002615) were synthesized by the Takara Bio, Inc. as described in detail [16–21]. Real-time reverse transcription polymerase chain reaction (RT-PCR) was performed using SYBR® *Premix Ex Taq™*II (Takara Bio, Inc.,) and LightCycler® (Roche Diagnostics, Mannheim, Germany). For internal control, 1043–1228 for human ß-actin mRNA (NM_001101) (Takara Bio, Inc.,) was used.

### PEDF concentration in culture medium

To quantify the degree of expression of PEDF by ARPE-19 cells, the cells were seeded in 96 well plates at 1.0 × 10^4^ cells/100 μL per well and were cultured with 5 to 25 μM of Aβ 1–40 for 48 h. The concentration of PEDF in the culture medium was measured with the human PEDF enzyme-linked immunosorbent assay (ELISA) kit (BioProducts MD, LLC., Middletown, US) according to the manufacturer’s protocol.

### RNA interference for receptor of advanced glycation end products (RAGE)

A small interfering RNA (siRNA) against RAGE was synthesized by Silencer® Select predesigned siRNA (Santa Cruz Biotechnology)*.* The target siRNA for RAGE, sc-36,374, and a human scrambled siRNA, sc-37,007, were purchased from Santa Cruz Biotechnology as control siRNA. Transfection of ARPE-19 cells by the siRNAs was performed according to the manufacturer’s protocol.

### Statistical analyses

The results are expressed as the means ± standard error of the means (SEMs). Student’s unpaired *t-*tests were performed to determine the statistical significance of the differences using the Graph Pad Prism software (GraphPad Software, La Jolla, CA).

## Results

### Exposure of ARPE-19 cells to 5 μM of Aβ 1–40 increases number of living ARPE-19 cells but exposure to 25 μM decreases number of living ARPE cells

**Fig. 1:**
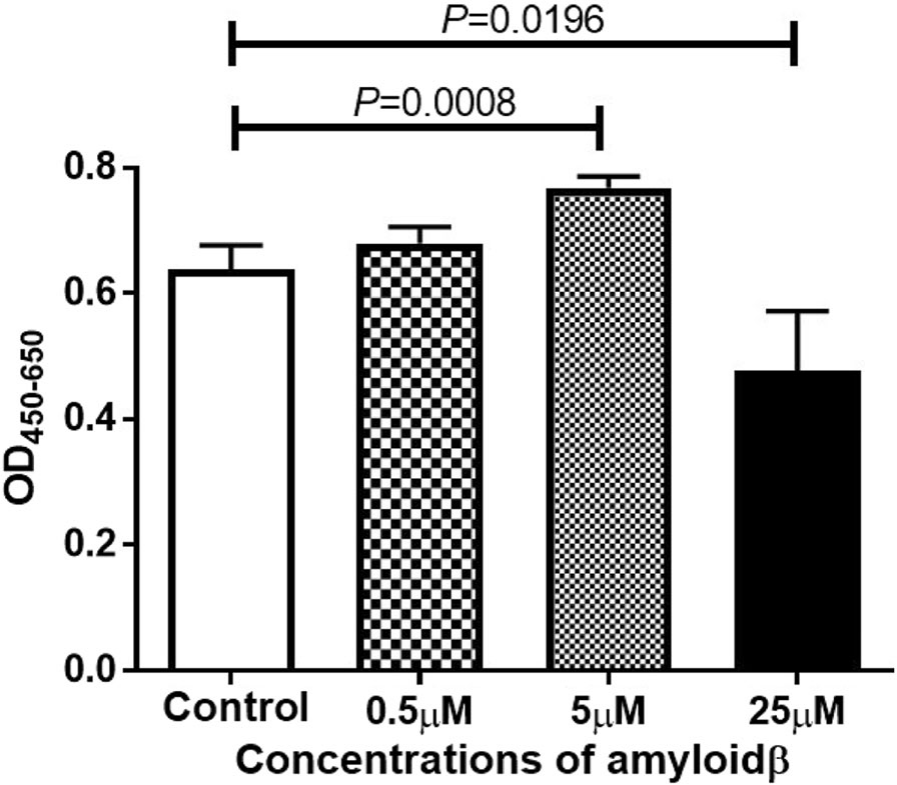
Effect of amyloid beta (Aβ) 1–40 exposure on the cells of a human adult retinal pigment epithelial cell (ARPE-19) line in culture. The ARPE-19 cells were incubated for 48 h with different concentrations of Aβ 1–40, and the number of living cells were determined by the WST-8 assay. The number of living cells was increased by exposure to 5 μM Aβ 1–40 but the number of living cells was decreased by 25 μM of Aβ 1–40 compared to the control group. Data are the means ± standard error of the means (SEMs) for each group (*n* = 4)

To evaluate the effects of Aβ 1–40 on the proliferation and death of ARPE-19 cells, the cells were exposed to different concentrations of Aβ 1–40 for 48 h. The number of living cells was determined by the WST-8 assay. The results showed that the number of living cells was significantly higher after exposure to 5 μM Aβ 1–40 than in the controls. However, the number of living cells was decreased significantly by exposure to 25 μM of Aβ 1–40 (Fig. 1).

### Exposure of ARPE-19 cells to 25 μM of Aβ 1–40 decreases replicative DNA synthesis and induces apoptosis of RPE cells

**Fig. 2:**
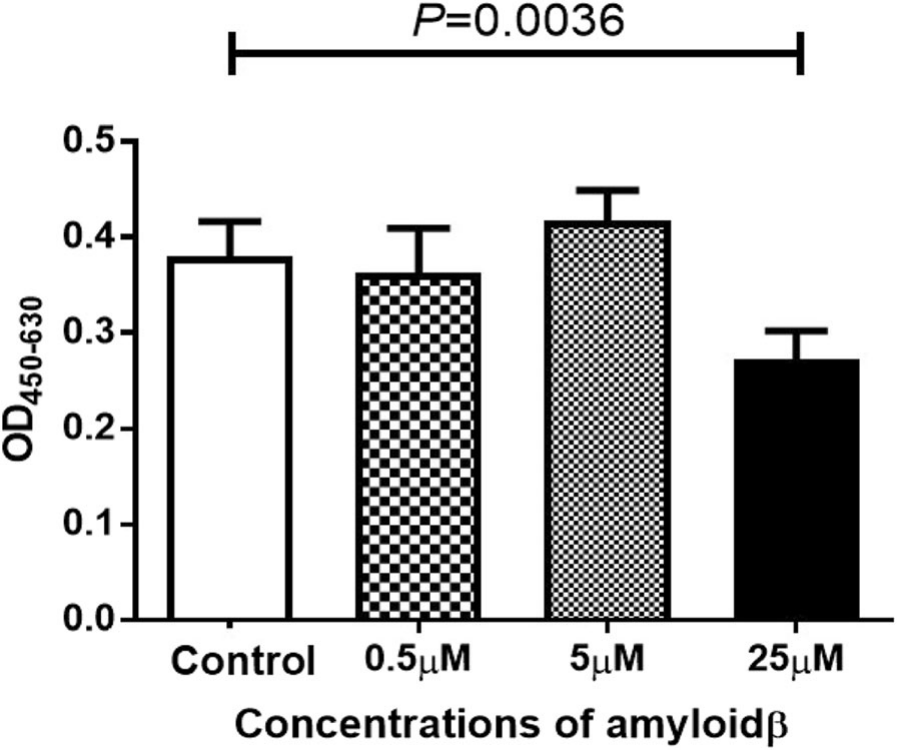
Effect of Aβ 1–40 on the DNA synthesis of ARPE-19 cells incubated for 24 h with Aβ 1–40. With 25 μM Aβ, there was a significant decrease in the DNA synthesis in the ARPE-19 cells. Data are the means ± SEMs for each group (*n* = 4)

The extent of replication of ARPE-19 cells was determined by the incorporation of BrdU. The replicative DNA synthesis by ARPE-19 cells exposed to 25 μM of Aβ 1–40 was significantly decreased (*P* < 0.04; 25 μM Aβ 1–40 vs control) indicating that 25 μM of Aβ 1–40 inhibited cellular proliferation (Fig. 2).

**Fig. 3:**
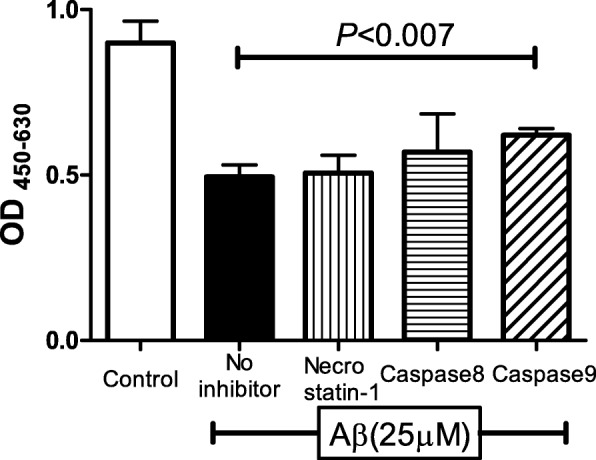
Apoptosis of ARPE-19 cells exposed to Aβ 1–40. The addition of a caspase-9 inhibitor suppresses the decrease in the number of living ARPE-19 cells exposed to 25 μM Aβ. This indicates that the apoptosis of ARPE-19 cell by 25 μM Aβ exposure was through the caspase-9 pathway. Data are the means ± SEMs for each group (*n* = 4)

Inhibitory assays for apoptosis and necroptosis were performed to determine whether Aβ increased the degree of apoptosis. The results showed that the addition of a caspase-9 inhibitor suppressed the decrease in the number of ARPE-19 cells exposed to 25 μM Aβ. This suggested that the addition of Aβ 1–40 leads to apoptosis of the RPE cells through the caspase-9 cascade. (Fig. 3).

On the other hand, exposure of the caspase-9 inhibitor to 5 μM of Aβ 1–40 increased the number of living ARPE-19 cells, but the difference in the replicative DNA synthesis between the control group and the 5 μM Aβ group was not significant. These results indicate that 5 μM of Aβ 1–40 suppressed the apoptosis of RPE cells.

### Exposure to Aβ 1–40 changes level of expression of mRNA of VEGF-A and PEDF in ARPE-19 cells

**Fig. 4:**
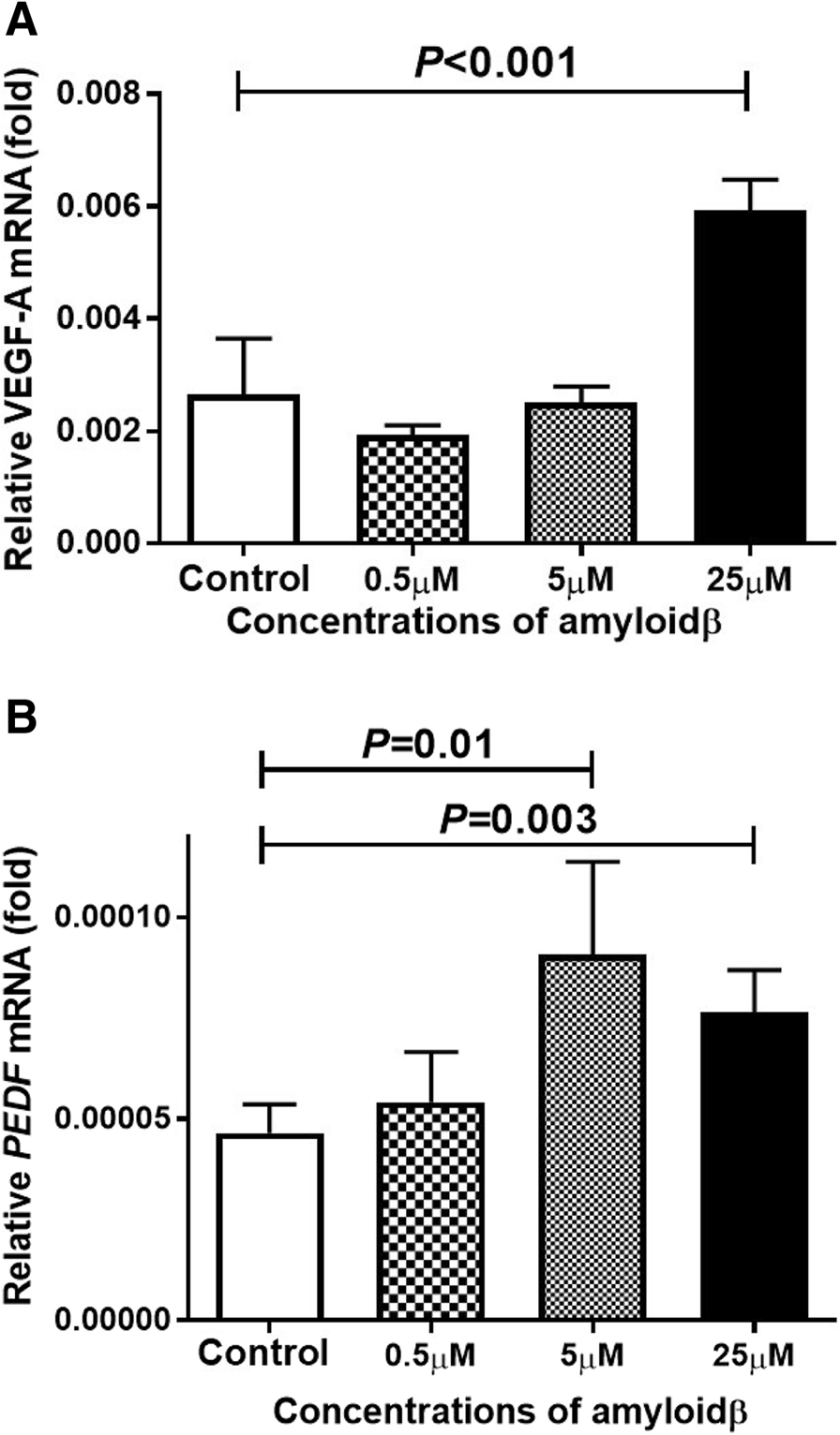
Induction of VEGF-A and PEDF expression in ARPE cells by exposure to Aβ 1–40. ARPE-19 cells were exposed to 25 μM Aβ 1–40 for 24 h, and the expressions of the mRNAs of *VEGF-A* and *PEDF* were determined by real-time RT-PCR using β-actin as an endogenous control. The level of the mRNA of *VEGF-A* is significantly increased only in the 25 μM Aβ group (A). On the other hand, the level of the mRNA of *PEDF* is increased by 5 μM Aβ 1–40 and is also increased by 25 μM Aβ 1–40 exposure (B). Data are the means ± SEMs for each group (*n* = 4)

It has been established that VEGF-A plays an important role in the pathology of AMD including RPE proliferation and choroidal neovascularization [22]. The level of expression of the mRNA of *VEGF-A* was determined by real-time RT-PCR. The results showed that the expression of *VEGF-A* mRNA was significantly increased only in the 25 μM Aβ 1–40 group (Fig. 4a).

PEDF was originally discovered in RPE cells, and it was found to play different roles and had anti-angiogenic, neuroprotective, and anti-apoptotic properties. We determined the expression of the mRNA of *PEDF* by real-time RT-PCR and found that the expression of the mRNA of *PEDF* in the ARPE-19 cells was increased after exposure to 5 μM Aβ 1–40 (*P* < 0.001 vs no addition). In addition, the levels of the mRNAs of *VEGF-A* and *PEDF* were also increased by prior exposure to 25 μM Aβ 1–40 (*P* < 0.001 vs no addition; *P =* 0.003 vs no addition respectively; Fig. 4b).

**Fig. 5:**
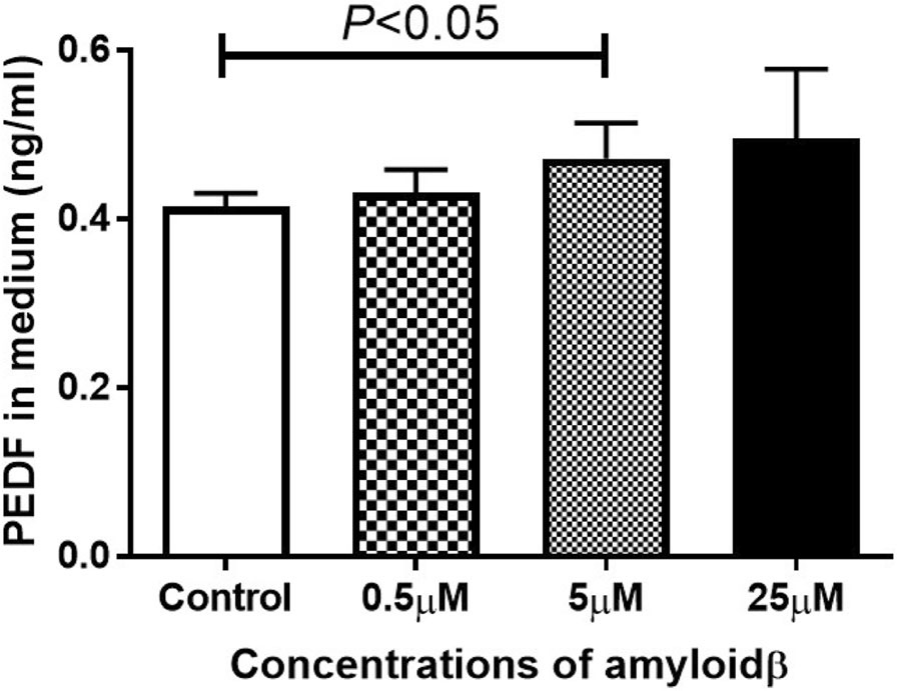
Concentrations of PEDF in the ARPE-19 cell culture medium determined by ELISA. ARPE-19 cells were exposed to Aβ 1–40 for 24 h. The concentration of PEDF is significantly increased only in the 5 μM Aβ 1–40 group. Data are the means ± SEMs for each group (*n* = 4)

Next, ELISA was performed to confirm that PEDF was secreted into the culture medium of the ARPE-19 cells. The secretion of PEDF was significantly increased after exposure to 5 μM Aβ 1–40 (*P* < 0.05 vs no addition; Fig. 5).

### Inhibition of PEDF signaling decreases cell proliferation

**Fig. 6:**
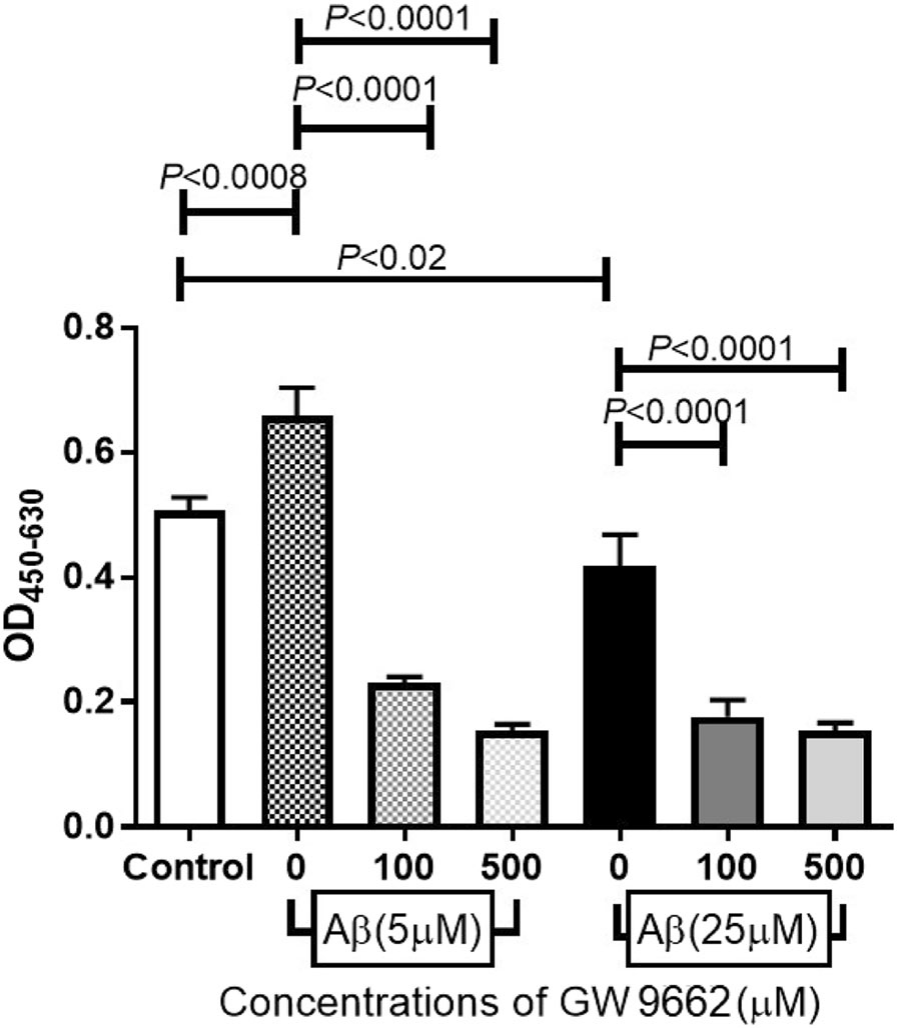
Inhibition of the PEDF signaling decreases cell proliferation. Aβ 1–40-exposed ARPE-19 cells were incubated with a PEDF intercellular inhibitor, GW9662. After 24 h, the number of living cells was measured by WST-8 cleavage. The number of Aβ 1–40-exposed ARPE-19 cells is significantly reduced by the combined addition of GW9662 and Aβ 1–40 compared to addition of Aβ 1–40 alone. Data are the means ± SEMs for each group (*n* = 4)

To determine whether PEDF exposure affected RPE cell proliferation, we added the PEDF receptor inhibitor, GW9662, to the Aβ 1–40-exposed ARPE-19 cells and measured the number of living cells with the WST-8 assay. The results showed that the number of living ARPE-19 cells exposed to Aβ 1–40 were significantly reduced by GW9662 (Fig. 6). This suggested that silencing the effect of PEDF decreased the number of living cells.

### Knockdown of RAGE by siRNA attenuated changes of VEGF, PEDF expression, and living cell numbers caused by Aβ

**Fig. 7:**
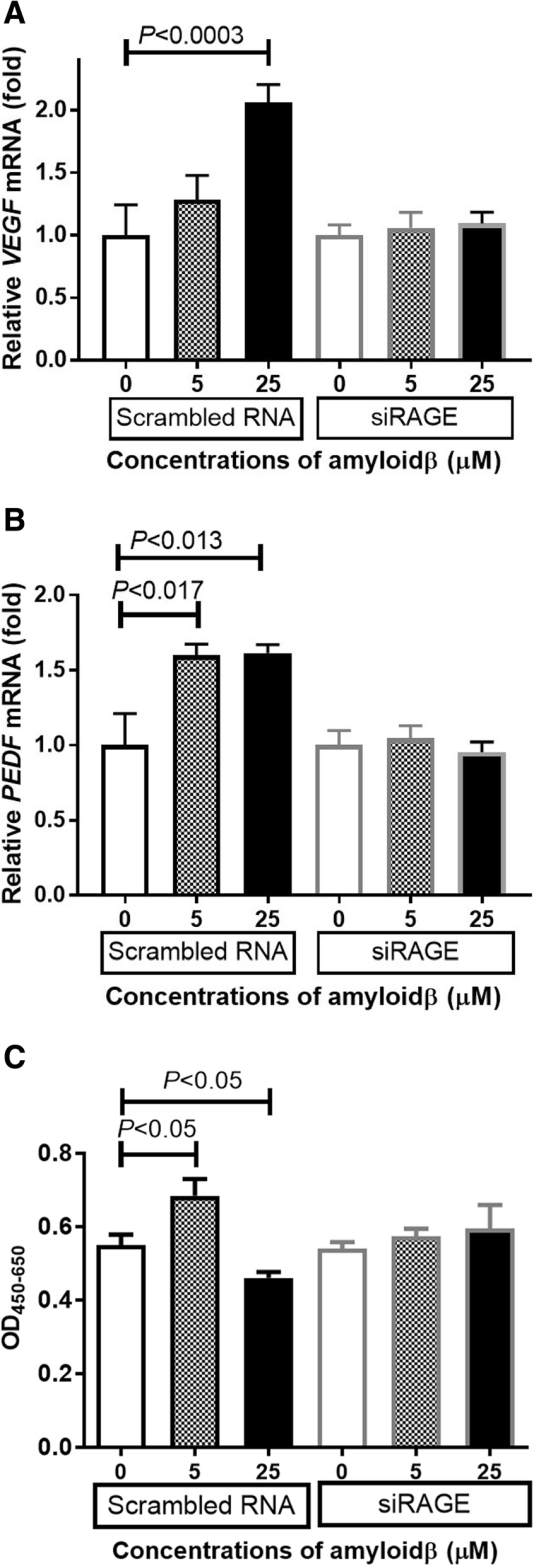
Relationship between RAGE and Aβ in the expression of VEGF and PEDF. *Si RAGE*-inserted ARPE-19 cells were incubated with Aβ 1–40 for 24 h, and the expression of the mRNA of *VEGF-A* and *PEDF* were measured by real-time RT-PCR using β-actin as an endogenous control. The control in each group was defined as 1 and show the number of relative comparisons in the experimental group. After 48 h of incubated with Aβ 1–40, the living cell number was measured by WST-8 assay. Knockdown of RAGE attenuated the increase and decrease of *VEGF* (**a**) and *PEDF* (**b**) expression caused by Aβ. In addition, Si-RAGE attenuated the increase and decrease of viable RPE cell number induced by Aβ addition (**c**). Data are the means ± SEMs for each group (*n* = 4)

It has been shown that Aβ can activate different signaling pathways and thereby activate a series of cell surface receptors. The results of several studies have shown that RAGE, a multi-ligand receptor for AGE, plays an important role as a receptor for Aβ. To determine whether RAGE was involved in the Aβ-stimulated RPE cell proliferation, we inserted a siRNA against *RAGE* into ARPE-19 cells, and then exposed them to Aβ 1–40. Our results showed that a knockdown of RAGE attenuated the increase and decrease of VEGF and PEDF expressions caused by the exposure to Aβ (Fig. 7a and b). In addition, Si-RAGE attenuated the change of viable RPE cell numbers induced by the addition of Aβ (Fig. 7c). These results indicated that Aβ caused a change in the viable cell number, and this stimulation is mediated mainly by RAGE.

## Discussion

The Aβs are 36–43 amino acid peptides that are associated with the pathogenesis of Alzheimer’s disease. It is well-known that amyloid plaques are specific findings in Alzheimer’s disease, and the plaques consist of several types of Aβ peptides. Aβ is produced from amyloid β precursor protein cleaved by β selectase or γ selectase [23]. The normal function of Aβ has not been determined although some experimental studies have demonstrated that the lack of Aβ does not cause any loss of physiological function [24]. However, there is also some evidence that Aβ has molecular protective activities including that against oxidative stress [25, 26]. On the other hand, Aβ is associated with the progression of other diseases, and the results of several studies showed that Aβ is involved in the pathogenesis of AMD including studies that used a molecular approach [27–29]. However, the functions of Aβ in human RPE cells have not been conclusively determined.

In this study, we had predicted that high concentrations of Aβ would be necessary to study the pathogenesis of AMD, and we used Aβ 1–40 that is relatively less toxic but more commonly distributed in drusen compared to other forms of Aβ. Our results demonstrated that the concentration of Aβ 1–40 regulated the expression of PEDF and VEGF in RPE cells in culture.

RAGE was originally found to be a receptor recognizing advanced-glycation end products (AGEs). RAGE consists of three superficial domains, and one of the domains recognizes AGEs [30]. After the activation of RAGE by binding to the AGEs, the AGE-RAGE pathway can lead to acute and chronic inflammatory disorders such as AMD [31]. In addition to the AGEs, there are other ligands that bind to RAGE. For example, Aβ and S100/calgranulins are also ligands of RAGE [32]. Because RAGE is also expressed on the surface of RPE cells and is increased under AMD conditions, we examined whether the Aβ-RAGE pathway is involved in the development of neovascular macular diseases with the expression of some cytokines.

Our results showed that the number of living RPE cell was significantly increased by exposure to 5 μM Aβ but markedly decreased by an exposure to 25 μM Aβ. These results indicated that the influence of Aβ on retinal pigment epithelial cells depends on its concentration. Yoshida et al. demonstrated that the concentration of VEGF in the conditioning medium of RPE cells exposed to Aβ increased in a dose dependent manner [33]. We confirmed that the expression of *VEGF-A* is increased in ARPE-19 cells after exposure to Aβ, and the mRNA of *VEGF* was elevated after exposure to higher concentrations of Aβ. Because these results cannot be explained by the change in the number of living cells, we also measured the concentration of PEDF in the culture medium. PEDF is a glycoprotein that belongs to the superfamily of serine protease inhibitors [34], and it has been shown to have a protective effect in retinal diseases especially age-related macular degeneration [35, 36]. Wang et al. reported that PEDF suppressed the apoptosis of RPE cells and the expression of inflammatory cytokines in a mouse AMD model [37]. In our study, the level of VEGF did not change but that of PEDF increased after exposure to 5 μM Aβ. Green et al. performed histopathologic studies of human CNVs, and they reported that CNVs were enveloped by proliferating RPE cells [38]. The proliferation of RPE cells in the 5 μM Aβ group may have been caused by the expression of PEDF, and it represents the proliferation of the surrounding pigment epithelial cells in eyes with age-related macular degeneration. PEDF has cellular protective effects even in the 25 μM Aβ group but the strong apoptosis signal in the 25 μ M group decreased the number of living cells. These findings are supported by the significant decrease in the number of living cells after exposure to GW 9662, an antagonist of PPAR-γ and a known nuclear inhibitor of PEDF [39].

These results predicted that apoptosis of RPE cells is high in the regions where Aβ deposits are found and VEGF enhances the development of CNVs in human eyes. On the other hand, when the number of Aβ deposits is low and RPE damage is slight, PEDF predominantly functions. Under these conditions, there is a protection and proliferation of the RPE cells.

Our results showed that the number of living cells was decreased by exposure to 25 μM of Aβ, and caspase-9 inhibition partially reduced the loss of living cells in the 25 μM Aβ group. Because caspase-9 is activated when apoptosis occurs through the caspase pathway, these results suggest that the apoptosis is mediated by caspase 9, and it is related to the death of retinal pigment epithelial cells caused by high concentration of Aβ. In addition, the siRNA knockdown study showed that Aβ probably acts by binding to RAGE. The Aβ-RAGE pathway can then lead RPE cells to caspase-9-related apoptosis.

This study was solely based on an in vitro approach in which RPE culture was the main component, and we mainly focused on molecular changes. While RPE dysfunction caused by Aβ is essential to the pathogenesis of AMD, choroidal endothelial cells may also play an important role [40]. But cross talk between the RPE cells and choroidal cells was not investigated in our study. In addition, we did not examine the functional assessments of the RPE cells including the RPE barrier or their phagocytic function. Thus, it is important to show the changes of the functional assessments of RPE. Further experiments are needed for understanding the pathogenesis of AMD.

According to a recent report [41], aducanumab, an anti-Aβ antibody, can reduce Aβ plaques in the brain of patients with Alzheimer’s disease. It has still not been tested whether this antibody is also effective on the Aβ deposits in the human retina. However, if the role played by amyloid β in age-related macular degeneration can be further determined, it may be possible that this drug could be used to treat eyes with AMD.

## Conclusions

The results showed that a low concentration of Aβ increases the level of PEDF and thus inhibits the apoptosis of RPE cells and increase the number of RPE cells. A high concentration Aβ induces RPE cell death through the caspase-9 cascade and enhances VEGF-A transcription in RPE cells. We suggest that this may lead to the development of choroidal neovascularization. The Aβ-RAGE pathway may lead to the expression VEGF-A and PEDF in ARPE-19 cells.
